# Anti-CRISPR proteins function through thermodynamic tuning and allosteric regulation of CRISPR RNA-guided surveillance complex

**DOI:** 10.1093/nar/gkac841

**Published:** 2022-10-10

**Authors:** Angela Patterson, Aidan White, Elizabeth Waymire, Sophie Fleck, Sarah Golden, Royce A Wilkinson, Blake Wiedenheft, Brian Bothner

**Affiliations:** Chemistry and Biochemistry Department, Montana State University, Bozeman, MT 59717, USA; Chemistry and Biochemistry Department, Montana State University, Bozeman, MT 59717, USA; Chemistry and Biochemistry Department, Montana State University, Bozeman, MT 59717, USA; Chemistry and Biochemistry Department, Montana State University, Bozeman, MT 59717, USA; Microbiology and Cell Biology Department, Montana State University, Bozeman, MT 59717, USA; Microbiology and Cell Biology Department, Montana State University, Bozeman, MT 59717, USA; Microbiology and Cell Biology Department, Montana State University, Bozeman, MT 59717, USA; Chemistry and Biochemistry Department, Montana State University, Bozeman, MT 59717, USA

## Abstract

CRISPR RNA-guided detection and degradation of foreign DNA is a dynamic process. Viruses can interfere with this cellular defense by expressing small proteins called anti-CRISPRs. While structural models of anti-CRISPRs bound to their target complex provide static snapshots that inform mechanism, the dynamics and thermodynamics of these interactions are often overlooked. Here, we use hydrogen deuterium exchange-mass spectrometry (HDX-MS) and differential scanning fluorimetry (DSF) experiments to determine how anti-CRISPR binding impacts the conformational landscape of the type IF CRISPR RNA guided surveillance complex (Csy) upon binding of two different anti-CRISPR proteins (AcrIF9 and AcrIF2). The results demonstrate that AcrIF2 binding relies on enthalpic stabilization, whereas AcrIF9 uses an entropy driven reaction to bind the CRISPR RNA-guided surveillance complex. Collectively, this work reveals the thermodynamic basis and mechanistic versatility of anti-CRISPR-mediated immune suppression. More broadly, this work presents a striking example of how allosteric effectors are employed to regulate nucleoprotein complexes.

## INTRODUCTION

**Figure 1.**
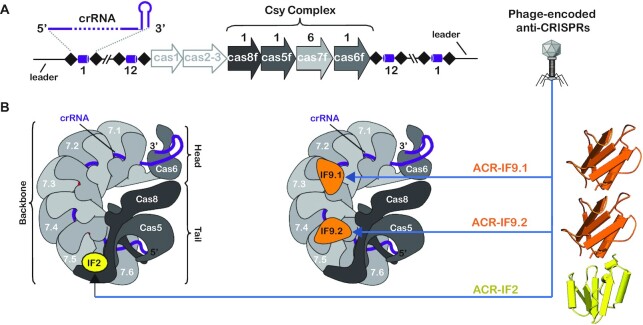
Cartoon depictions of the Csy CRISPR interference complex and its interactions with AcrIF9 and AcrIF2. (**A**) In the Type IF CRISPR system, the Csy complex consists of four cas genes (cas6f–cas8f, grey) and the crRNA (purple) is coded for by the CRISPR locus (black/purple). (**B**) Cartoon depictions of the Csy complex either bound to AcrIF2 (left, yellow) or AcrIF9 (right, orange). One AcrIF2 binds at the tail of the complex, interacting with Cas7.6 and Cas8f, and two AcrIF9 proteins bind to the Csy complex along the Cas7 backbone.

CRISPR (clustered regularly interspaced short palindromic repeats) and associated *cas* genes are essential components of adaptive immune systems that protect bacteria and archaea from foreign genetic elements (1–4). These systems are diverse and as such are classified into two classes, six types and multiple sub-types (4,5). The Class 1 CRISPR systems are characterized by a multi-subunit surveillance complex, which is formed when multiple Cas subunits assemble around the processed CRISPR RNA (crRNA) (4–7). In type IF systems, the CRISPR surveillance complex is called Csy (or IF-Cascade) (8,9). It consists of crRNA surrounded by a Cas6f protein at the head of the complex, six Cas7 subunits, which form the backbone or torso, and a heterodimer of Cas5 and Cas8f that form the tail (Figure 1, Supplemental Figure S1) (9–13). This complex patrols the intracellular environment for a complementary DNA target (6,14). DNA surveillance is a multi-step process that involves electrostatic mediated non-sequence specific interactions, protein mediated detection of a short (2–5 nt) protospacer adjacent motif (PAM), and crRNA-guided strand invasion, which involves base pairing that extends from one end of the crRNA-guide to the other (15,16). Recognition of target dsDNA by the surveillance complex drives a conformational change that exposes a binding site for recruitment of the Cas3 nuclease-helicase, which processively degrades the invading DNA (16–25).

To escape CRISPR-mediated immunity, viruses have evolved small (<150 amino acid) anti-CRISPR (Acr) proteins that suppress CRISPR immune function (26–28). A variety of mechanisms have been discovered and described for Acr mediated inhibition of CRISPR systems (26,29–32). Of the currently characterized IF Acrs, there appear to be two main mechanisms of Csy complex inhibition: prevention of substrate binding or prevention of Cas2/3 recruitment (26,33). Of these two inhibition regimes, the prevention of dsDNA binding occurs via sterically blocking the crRNA from interactions with a complementary target (e.g. AcrIF1, AcrIF9 and AcrIF14), or blocking recognition of the PAM (e.g. AcrIF2, AcrIF6-8, AcrIF10 and AcrIF11) (10,26,31,33,34). While both strategies prevent targeted elimination of dsDNA, we were interested to see if there were similarities or discrepancies in the underlying energetics that drive each type of inhibition. To test this, we chose to study the interactions between the Csy complex and either AcrIF9 or AcrIF2. Two molecules of AcrIF9 (AcrIF9.1 and AcrIF9.2) bind to two Cas7 subunits (Cas7.4f and Cas7.6f) in a way that sterically blocks access of the DNA target to the crRNA-guide. In contrast, AcrIF2, a DNA mimic, has a structure and electrostatic surface potential reminiscent of a PAM containing dsDNA target (31). Driven by interactions between acidic residues on the Acr and lysine rich regions of Cas7.6 and Cas8f, AcrIF2 specifically binds to a PAM recognition domain located in the N-terminus of the Cas8f subunit (26,31).

Structural models of Csy bound by AcrIF2 and AcrIF9 provide significant detail on the location of binding and the mechanisms of CRISPR suppression (31,34). While both Acr bound Csy complexes are similar in structure, there are conformational changes which occur distal to the Acr binding site (Supplemental Figure S1). Though these conformational changes are not large (RMSD between structures is <6 Å), the presence of long-distance changes indicates that allostery may be involved in Acr function. This idea is supported by our previous work on the type IE CRISPR surveillance complex (i.e. Cascade), which demonstrated a role for allostery in nuclease recruitment (35). Here, we set out to determine if allostery plays a role in Acr function.

Allostery can cause changes in either structure or dynamics upon ligand binding (36). Ligand binding alters the energy landscape of the protein such that either there is a change in the overall most stable conformation or there is a change in the rate of interconversion between conformations (37). By comparing the structure and dynamics of ligand bound and unbound forms, functional properties of the conformational ensemble, as well as the basis for Csy allostery can be elucidated. Here, we use hydrogen deuterium exchange coupled to mass spectrometry (HDX-MS) and differential scanning fluorimetry (DSF) to examine the protein stability and dynamics of Acr binding to the Csy complex. We present a model for Csy inhibition by AcrIF9 and AcrIF2 in which allosteric effects are driven by entropic or enthalpic modulation, respectively. Our model is consistent with recent ensemble theories of allostery and highlights that these two Acrs function through fundamentally different mechanisms.

## MATERIALS AND METHODS

### Purification of the Csy complex

The Csy complex was purified as described previously (9,38). Csy genes and a synthetic CRISPR were co-expressed on separate vectors (Addgene IDs: 89232 and 89244) in *Escherichia coli* BL21 (DE3) cells (NEB). Expression was induced with 0.5 mM isopropyl-d-1-thiogalactopyranoside (IPTG) at an optical density (OD_600_ nm) of 0.5. Cells were incubated overnight at 16°C, then pelleted by centrifugation (3000 × g for 10 min at 4°C) and re-suspended in lysis buffer containing 50 mM Tris at pH 7.5 and 500 mM sodium chloride. Pellets were sonicated on ice and the lysate was clarified by centrifugation at 10 000 × g for 25 min at 4°C. The Csy complex was affinity purified using Ni-NTA resin (QIAGEN). The resin was washed with five column volumes of lysis buffer before elution with 50 mM Tris (pH 7.5), 300 mM NaCl, 250 mM imidazole, 5% glycerol. Protein was then concentrated (Corning Spin-X concentrators) at 4°C prior to size-exclusion chromatography (Superdex 200, GE Healthcare) in 20 mM HEPES, pH 7.5, 100 mM NaCl, 5% glycerol and 1 mM TCEP (SEC buffer).

### Purification of the ssDNA bound Csy complex

An 80-nt ssDNA with 32-nts (bold) complementary to the crRNA-guide (GCT GTA CGT CAC TAT CGA AGC AAT A**CA GGT AGA CGC GGA CAT CAA GCC CGC CGT GAA** GGT GCA GCT TCT CTA CAG AGT GC) was incubated at a 3-fold molar excess with the Csy complex for 15 min at 37°C. The DNA-bound Csy was separated from the unbound DNA by size-exclusion chromatography (Superdex 200, GE Healthcare) performed in SEC buffer.

### Purification of Csy–AcrIF9 complex

The Csy–AcrIF9 complex was produced by co-transforming *E. coli* BL21 DE3 cells with a plasmid containing the AcrIF9 gene, along with the two plasmids for expression of the Csy complex. Expression was induced with 0.5 mM isopropyl-d-1-thiogalactopyranoside (IPTG) at an optical density (OD_600_ _nm_) of 0.5. The Csy–AcrIF9 complex was purified from lysates using Ni-affinity chromatography followed by size-exclusion chromatography (Superdex 200, GE Healthcare) performed in SEC buffer.

### Purification of AcrIF2–Csy complex

The AcrIF2–Csy complex was purified as previously described (9). Briefly, AcrIF2 (Addgene plasmid #89234) was overexpressed in *E. coli* BL21 DE3 cells grown to an OD_600_ of 0.4 and then induced with IPTG at 16°C for 16 h. Cells were collected by centrifugation and suspended in a lysis buffer containing 50 mM Tris, pH 7.5, 500 mM NaCl. The cells were lysed by sonication and the lysate was centrifuged at 10 000 × g for 25 min to remove cell debris. The supernatant was added into a Ni-NTA column, washed with lysis buffer supplemented with 20 mM imidazole and eluted using a using a linear gradient of imidazole (20–300 mM). Fractions were collected and concentrated (Corning Spin-X concentrators) at 4°C. The purification tag was removed by TEV protease cleavage in SEC buffer (20 mM Tris, pH 7.5, 100 mM NaCl, 1 mM TCEP, 5% glycerol). The cleaved AcrIF2 was separated from the His-tag and uncleaved product by passing through a Ni-NTA column. A 4-fold molar excess of the cleaved AcrIF2 was mixed with purified Csy complex and incubated at 37°C for 15 min. Free Acr protein was separated from the Csy–AcfIF2 complex using a Superdex 200 size-exclusion column (GE Healthcare) in SEC buffer.

### Intact hydrogen deuterium exchange mass spectrometry

Concentrated protein stocks, Csy (9.7 μM), Csy with ssDNA (15.7 μM), Csy with AcrIF9 (13.7 μM), and Csy with AcrIF2 (35.7 μM) were diluted 1:10 into deuterated 50 mM HEPES 150 mM KCl, pH 7.5 or into non-deuterated buffer for the 0-min controls. The reaction was placed in an Agilent 1290 UPLC series LC containing an autosampler and 10 μl were withdrawn at 1, 8, 60, 180 and 1,440 min. LC analysis was conducted using a Phenomenex Onyx Monolithic C18 reverse phase column (100 × 2 mm) at 1°C using a flow rate of 300 μl/min under the following conditions: 1.0 min, 10% B; 1.0−4.0 min, 10−70% B; 4.0−4.5 min, 70–90% B; 4.5–6.0 min, 10% B; solvent A = 0.1% FA (Sigma) in water (ThermoFisher) and solvent B = 0.1% formic acid (FA) in acetonitrile (ACN) (ThermoFisher), or a Phenomenex Luna C5 column (30 × 2 mm), 1°C at 200 μl/min with the following gradient: 0 min, 10% B; 1.0−6.0 min, 10−90% B; 6.0–7.0 min, 10% B. The LC system was coupled to a microTOF mass spectrometer (Bruker Daltonics). Data for both experiments were acquired in positive mode. Electrospray settings were as follows: nebulizer set to 5.0 bar, drying gas at 7.0 l/min, drying temperature at 200°C, and capillary voltage at 4.5 kV. The capillary exit was set at 150 V, skimmer 1 at 60 V, hexapole 1 at 26V, hexapole RF at 350 V_pp_, and skimmer 2 at 22V. Data analysis was carried out using Bruker DataAnalysis with the MaximumEntropy plug-in. Each reaction was performed in triplicate. Statistically significant differences were determined using a two tailed Student's *t*-test with a *P* < 0.05 being considered significant. Apo Csy was used as a baseline to normalize exchange patterns between conditions.

### Peptide level hydrogen–deuterium exchange mass spectrometry

As described for intact HDX-MS, protein was diluted 1:10 into deuterated buffer. Ten microliters were removed from the reaction mixture at 0.5, 3, 30, 180 and 1800 min and quenched by diluting 1:6 into 0.75% formic acid (FA, Sigma) and 0.25 mg/ml porcine pepsin (Sigma) at pH 2.5 on ice. The sample was then digested for 2 min on ice with vortexing every 30 s. After digestion, the samples were flash frozen and stored in liquid nitrogen until LC–MS analysis. Each reaction was performed in triplicate.

LC–MS analysis was carried out as described in Patterson *et al.* (39). Briefly, the analysis was completed on a 1290 UPLC series chromatography stack (Agilent Technologies) coupled directly to a 6538 UHD Accurate-Mass QTOF LC/MS mass spectrometer (Agilent Technologies). Before electrospray−time-of-flight (ESI-TOF) analysis, peptides were separated on a reverse phase (RP) column (Phenomenex Onyx Monolithic C18 column, 100 × 2 mm) at 1°C using a flow rate of 500 μl/min under the following conditions: 1.0 min, 5% B; 1.0−9.0 min, 5−45% B; 9.0−11.8 min, 45–95% B; 11.80−12.0 min, 5% B; solvent A = 0.1% FA (Sigma) in water (ThermoFisher) and solvent B = 0.1% FA in ACN (ThermoFisher). Data were acquired at 2 Hz over the scan range 50−1700 *m*/*z* in positive mode. Electrospray settings were as follows: nebulizer set to 3.7 bar, drying gas at 8.0 l/min, drying temperature at 350°C, and capillary voltage at 3.5 kV. Data analysis was carried out as previously described (35,40) using MassHunter Qualitative Analysis (Agilent Technologies), Peptide Analysis Worksheet (PAWs, ProteoMetrics LLC), SearchGUI v3.3.16 (41), PeptideShaker v 1.16.42 (42), HDExaminer v 2.5.1 (Sierra Analytics) and visualized using UCSF Chimera (43).

### Differential scanning fluorimetry

Differential scanning fluorimetry was performed with a final reaction volume of 30 μl. Four experimental conditions were tested: one with Csy only (final concentration of 0.323 μM), one with AcrIF9-bound Csy (final concentration of 0.457 μM), one with AcrIF2-bound Csy (final concentration 0.475 μM), and another with ssDNA-bound Csy (final concentration of 0.523 μM). Each reaction (*n* = 3) was buffered in 100 mM citrate phosphate buffer (pH 7.5) and 2.5 μl of 50× stock SYPRO orange dye (Invitrogen). Protein only and dye only controls were also tested. The samples were then loaded into the Rotor-Gene Q instrument (Qiagen) reading SYPRO orange absorbance at 570 nm as temperature was increased from 25°C to 95°C at 1°C/min.

## RESULTS

Conformational changes are known to be critical to CRISPR function (20,21,35,44), and the structural models of Acr bound Csy complexes reveal small differences in conformation compared to the unbound complex (Supplemental Figure S1) (31,34). These results led us to hypothesize that anti-CRISPRs allosterically alter the stability and dynamics of the crRNA-guided surveillance complex. To test this hypothesis, we used HDX-MS to measure the strength and stability of the peptide backbone hydrogen bonding network, while DSF was used to measure thermal stability with or without AcrIF9 or AcrIF2 bound.

### Intact protein HDX-MS shows global differences in hydrogen exchange upon Acr binding

**Figure 2.**
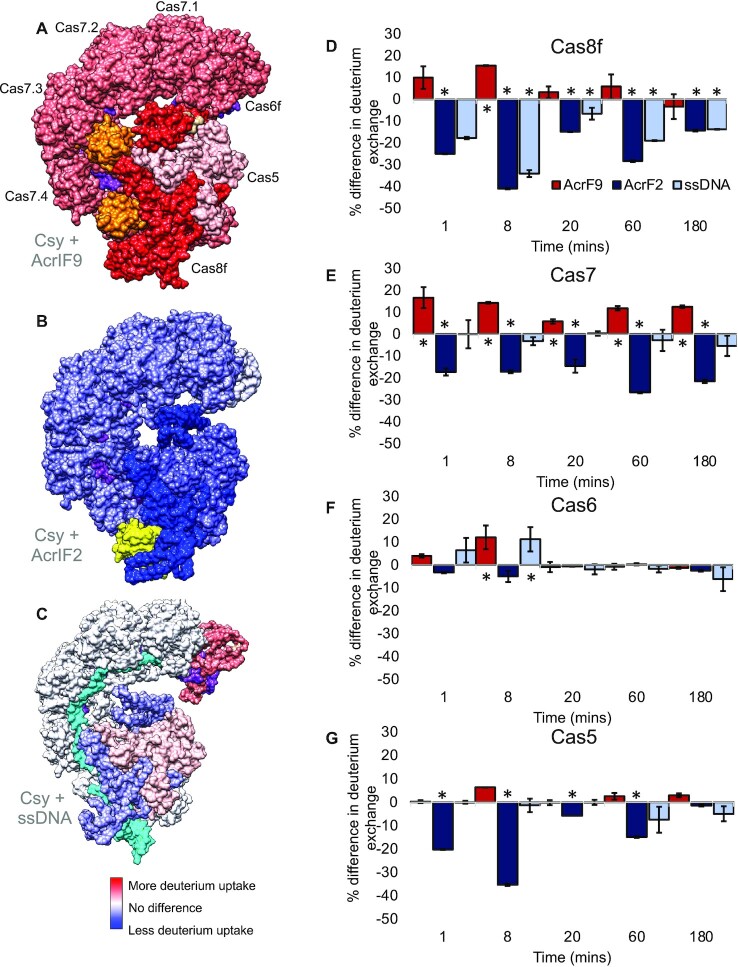
Protein level HDX shows differences in dynamics of the Csy complex when different anti-CRISPRs or ssDNA are bound. (**A**) Intact HDX data from the 8-minute time point mapped onto the AcrIF9 bound structure of Csy, (**B**) the AcrIF2 bound structure of Csy and (**C**) the DNA bound structure of Csy. Subunits in red show more deuterium exchange when either Acr or ssDNA is bound, while those in blue show less deuterium exchange in the bound form. Proteins in white show no difference in exchange. Acrs are shown in orange (AcrIF9) and yellow (AcrIF2), DNA in cyan, and crRNA is shown in purple. (**D**) The difference in deuterium uptake in either the AcrIF9 (red), AcrIF2 (dark blue) or ssDNA bound condition (light blue) compared to unbound Csy for the Cas8 subunit. Positive values indicate an increase in deuterium exchange when either the Acr or ssDNA is bound, negative values indicate a decrease in exchange. Error bars represent the standard deviation of three replicates. * indicates a significant *P*-value <0.05. (**E**) Differences in deuterium exchange for the Cas7 subunit. Colors are the same as panel (D). AcrIF9 bound Csy shows a more dynamic Cas7 backbone while the AcrIF2 bound Csy shows a Cas7 backbone with a more stable secondary structure. (**F**) Differences in deuterium exchange for the Cas6 subunit. (**G**) Differences in deuterium exchange for the Cas5 subunit.

To measure changes in HD-exchange of the Csy complex on a global scale, intact protein HDX-MS was performed on the unbound complex as well as Csy bound to either AcrIF9 or AcrIF2. A solution of concentrated complex was diluted in deuterated buffer and exchange was monitored from one to 1440 min. The reaction was quenched by injection into the acidic solvent stream of the LC–MS system. Deuterium uptake for each subunit of the Csy complex was calculated from the deconvoluted protein mass at each timepoint. Quantitative differences in deuterium uptake between conditions were calculated and mapped onto the structure (Figure 2A and B).

The intact protein HDX-MS showed differences in exchange between the AcrIF9 and the AcrIF2 bound forms of the Csy complex. In the AcrIF9 bound form, we measured no significant difference in deuterium exchange at the head (Cas6f, except for the 8-min time point) or tail (Cas5 and Cas8f except for the 8-min time point) of the Csy complex compared to the unbound form. However, there was a significant increase in exchange in Cas7 located in the backbone of the complex (*P* < 0.05 from 1 to 60 min, Figure 2E). Because the Cas7 subunits have an identical amino acid sequence, the differences in deuterium exchange reported cannot be assigned to an individual Cas7 subunit and should be taken as a population average. The average Cas7 population continues to exchange throughout the HDX time course (with the exception of the AcrIF9 bound condition where maximum observed deuteration occurs by the 3-h time point (Supplementary Data Set)). In the AcrIF2 bound form of Csy, we observed no significant difference in deuterium uptake at the head of the complex (Cas6f, Figure 2F). There was a significant decrease in deuterium exchange in the tail of the complex (Cas5 early time points and Cas8f at all time points Figure 2G and D) and the backbone (Cas7, all time points are statistically significant, Figure 2E). The decrease in deuterium exchange is present throughout the time course in Cas7 and is most pronounced at early time points in Cas8.

### Peptide level HDX-MS highlights localized differences in protein dynamics upon Acr binding

**Figure 3.**
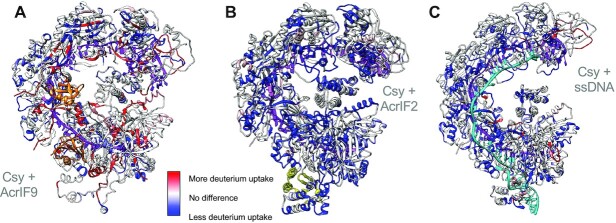
Peptide level HDX shows differences in dynamics of the Csy complex when different ligands are bound. Peptide level HDX data from the 180-min time point mapped to the AcrIF9 (**A**), AcrIF2 (**B**) or DNA (**C**) bound structures. Areas in red show more deuterium exchange when the indicated ligand is bound, while areas in blue show less deuterium exchange in the bound condition. Regions in white either have no coverage or show no difference in exchange. Acrs are shown in orange (AcrIF9) or yellow (AcrIF2), DNA in cyan, and crRNA is shown in purple. AcrIF9 causes a complex change in dynamics of the Csy complex when bound while AcrIF2 and ssDNA show a net stabilization of the secondary structure of the Csy complex when bound, though this trend is more pronounced in the AcrIF2 bound form than the ssDNA bound form.

To gain a higher-resolution view of the changes in deuterium exchange, peptide level HDX-MS was performed on the Csy complex alone and bound to AcrIF9 or AcrIF2. A solution of concentrated complex was diluted into deuterated buffer, as above, and allowed to exchange for 0.5–1440 min. The reaction was quenched with formic acid followed by pepsin digestion and LC–MS analysis. The Csy ribonucleoprotein complex totaling >1300 amino acid residues over four different proteins pushes the limits of complexity for peptide level HDX-MS due to the challenge in making unequivocal peptide assignments. We included in our analysis only Csy peptides that (i) could be uniquely assigned based on accurate mass and MS/MS sequence tags, (ii) were found in all three replicates of all five time points and (iii) were observed in each of the compared conditions. In total, we tracked exchange in >100 peptides (Supplemental Figure S2) providing coverage from the head to the tail of the complex. For Cas7 there was >60% sequence coverage. Deuterium uptake per peptide was calculated and used to generate butterfly plots showing exchange across the protein sequence (Supplemental Figure S3). The peptide-level differences in deuterium uptake between the Csy complex alone (Supplemental Figure S4) and when bound by AcrIF9 or AcrIF2 were mapped on to the structure (Figure 3A and B).

The AcrIF9 bound form of Csy reveals regions of increased deuterium uptake in peptides that are present along the length of the crRNA and decreased deuterium uptake in regions up to and beyond 75 Å from the binding site (30 Å from the crRNA) (Figure 3A, Supplemental Figure S3). The increase in exchange is highest in the Cas7 backbone. While some of the increases in exchange are modest, many of these regions show an increase in deuterium uptake of over 10% at multiple time points. Of the Cas7 peptides analyzed, only three showed bimodal uptake behavior (99–113, 235–248 and 285–312; Supplemental Figure S5). All other peptides (across all subunits) showed unimodal isotopic distributions. This indicates that the increase in exchange is nearly uniform in each of the Cas7 subunits and not restricted to a subset of subunits directly interacting with AcrIF9.

The AcrIF2 bound Csy peptide exchange profile differed dramatically from the AcrIF9 bound complex (Figure 3B). In accord with the intact HDX-MS data which displayed net protection from exchange across the Csy complex, the prevailing outcome for the AcrIF2–Csy complex peptide exchange was a significant decrease in exchange across the complex at all time points with only a few peptides showing increased exchange upon AcrIF2 binding (Supplemental Figure S3). The decrease in exchange was >10% for most peptides and in many cases occurs distal from the AcrIF2 binding site.

### ssDNA binding decreases HDX throughout the Csy complex

Although AcrIF2 mimics DNA recognized by the tail of the Csy complex (Cas7–8 ‘vise’), AcrIF2 binding does not facilitate the backbone extension observed upon crRNA:DNA hybridization (Supplemental Figure S6). Instead, the AcrIF2–Csy complex more closely resembles the unbound Csy complex. This prompted us to ask if the structural models may be missing important biophysical differences in the stability or dynamics of the different forms of Csy. For the following experiments, a single stranded DNA (ssDNA) bound version of Csy was used. Our reasoning for using a ssDNA target was that binding would induce the associated conformational changes that are primarily associated with backbone extension, without the exchange pattern being confounded by the rotation of Cas8f and non-target strand (displaced strand) DNA binding on the surface of Csy (10). Therefore, any observed protection from deuterium exchange would not be caused by shielding of solvent by the displaced strand.

Intact protein and peptide level HDX-MS was performed on the Csy complex bound to ssDNA. In the intact HDX-MS, we measured a significant decrease in exchange for Cas8f after binding a complementary ssDNA target (Figure 2C and D). The difference in exchange between the ssDNA bound Csy complex and the unbound Csy complex is statistically significant at the 8-minute time point and beyond. In this instance, the Cas8f subunit takes up less deuterium after binding ssDNA. In the Cas7 backbone of the complex we also observed a slight decrease in exchange (Figure 2C and E). Because a structure of Csy bound to ssDNA is currently unavailable, the partially duplexed dsDNA bound Csy complex structure (PDBID: 6B44) was used in the analysis. While both the partially and completely dsDNA bound Csy complexes show an ∼20 Å elongation, the overall conformation of the Cas7 subunits changes little when the partially dsDNA substrate is bound (10,20). This could explain the modest difference observed in ssDNA bound Csy compared to the unbound complex within the intact HDX-MS data.

Peptide level HDX clarified the behavior as there were areas of increased deuterium exchange upon ssDNA binding. However, the majority of peptides analyzed showed a decrease in exchange when compared to unbound Csy (Figure 3C). In particular, the hook domain of Cas8f (residues 1–166), had peptides that displayed increased deuterium uptake as well as peptides that became protected from exchange upon ssDNA binding. The differences in deuterium uptake profiles within this region are supported by the conformational change observed in the structure of the dsDNA bound Csy complex. This domain adopts an ‘open’ conformation in the unbound Csy complex, transitioning to a closed conformation upon productive binding of the dsDNA substrate (10). Interestingly, peptides spanning residues 66–72 (all time points) and 135–150 (early time points) showed a modest increase in deuterium exchange when ssDNA is bound (Supplemental Figure S7A). This change in deuterium uptake indicates this region is altered upon binding of the target strand of DNA. Comparisons between the ssDNA bound complex and the AcrIF2 bound complex in this region showed similar protection from deuterium exchange in three peptides: residues 4–20, 56–65, 145–150 (Supplemental Figure S7B). The net protection from exchange in the Cas8f and Cas7 subunits of the ssDNA bound complex is redolent of the protection from exchange observed in the AcrIF2 bound form of Csy, however, AcrIF2 protection is generally more significant.

### Differences in Csy thermal stability upon Acr binding

**Figure 4.**
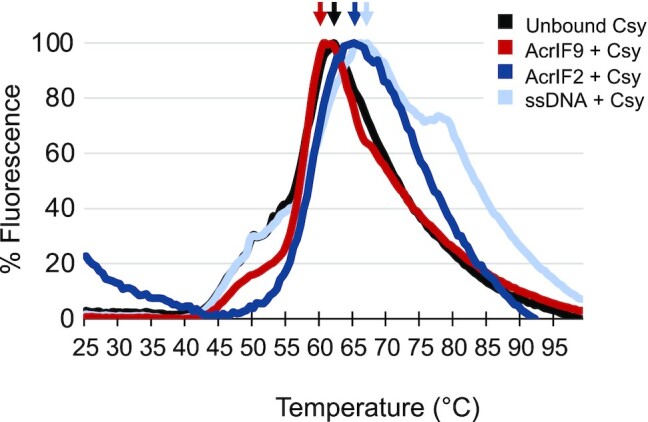
Differential scanning fluorimetry was used to probe the thermal stability of the ligand bound Csy complexes. Differential scanning fluorimetry data for the unbound (black), ssDNA (light blue), AcrIF9 (red), and AcrIF2 bound (dark blue) Csy complex. Arrows show the maximum of each peak which is used to compare the forms. The ssDNA and AcrIF2 bound conditions show a thermal stabilization compared to the unbound Csy complex, whereas the AcrIF9 bound condition shows no significant change in thermal stability when compared to the unbound Csy condition. Differences in the temperature profiles of AcrIF2 and AcrIF9 indicate that these ligands have differing effects on Csy complex thermal stability.

The HDX data shows changes in deuterium exchange in the AcrIF9 and AcrIF2 bound forms of Csy. This reveals differences in the conformational stability and/or dynamics. The most straight-forward explanation for this is that the two anti-CRISPRs have different modes of binding. This prompted us to use an orthogonal test of complex stability. The thermal stability of the AcrIF9 bound, AcrIF2 bound, ssDNA bound and unbound Csy complexes was measured using Differential Scanning Fluorimetry (DSF) (Figure 4). The resulting fluorescence and temperature data were used to construct a melting curve to compare protein thermal stability between conditions. The maximum intensity of each curve was used as a point of reference (Figure 4, arrows). Using unbound Csy as a benchmark, the ssDNA bound form has a notably right-shifted curve (4°C higher), showing that ssDNA binding thermally stabilizes the Csy complex (Figure 4, arrows). AcrIF9 bound Csy, on the other hand, is similar to unbound Csy indicating that the global thermal stability of the complex is unaltered upon AcrIF9 binding. However, the AcrIF2 bound form shows an increase in temperature at the maximum intensity compared to unbound Csy (3°C difference), indicating that the Csy complex becomes thermally stabilized upon AcrIF2 binding. The increased thermal stability of AcrIF2 bound Csy parallels the increased conformational stability measured in the HDX-MS experiments.

The presence of shoulders on a melting curve rules out a simple two state system and is indicative of local unfolding events (45). In the AcrIF9 bound complex, the shoulder at 50°C is less pronounced than unbound and ssDNA bound conditions. In the AcrIF2 complex, the shoulder is absent, indicating that the contributing protein region has been stabilized. The lack of a shoulder and the symmetric shape of the curve suggest that the AcrIF2 bound Csy complex unfolds cooperatively. There is a shoulder in the ssDNA bound Csy complex at ∼80°C, which is well above the maximum intensity (Tm). The ssDNA bound form of Csy is the only one that has a distinct shoulder above the Tm, which implies that a component remains folded even after most of the complex melts. Because the dye used in these experiments is not known to interact with nucleic acid duplexes, the high temperature shoulder is presumed to report on Csy releasing from ssDNA (46).

## DISCUSSION

Protein-protein interactions are ultimately driven by free energy. This is formalized in the equation for Gibbs free energy (G) as, Δ*G* = Δ*H* – *T*Δ*S*, where *H* is enthalpy, *T* temperature and *S* is entropy. A reaction is favorable when the change in free energy (Δ*G*) is negative. Thus, protein-protein interactions are a balance of enthalpic and entropic contributions. At a fundamental level, HDX provides a readout of the stability of hydrogen bonding networks, allowing it to be used as a proxy for stability and dynamics when investigating a protein under different conditions (47,48). When used judiciously, it can provide qualitative insight into the entropic and enthalpic contribution to binding. In a similar manner, thermal stability determined by DSF can be used to assess the relative enthalpic contribution to protein binding.

### AcrIF9 binding is entropically stabilized

In an HDX time-course, exchange at early time points (seconds) report mostly on solvent accessibility and high frequency low amplitude motion (49). Exchange at time points on the minutes to hours scale, implies lower frequency lapses in the H-bond network consistent with slow conformational change and/or protein dynamics (50,51). In the case of Cas7, we observed an increase in deuterium exchange in the AcrIF9 bound form across all time points (Figure 2). This suggests, on average, each Cas7 subunit is more solvent accessible and less stable in the AcrIF9 bound complex. A decrease in stability would mean an increase in entropy for the complex which would contribute to making AcrIF9 binding favorable.

In a protein complex, entropy and enthalpy are in a delicate balance. As the entropy increases, the hydrogen bonding network present within the protein secondary, tertiary, or quaternary structure weakens, leading to a less favorable enthalpy of binding. Therefore, if there is an increase in entropy in Csy upon binding of AcrIF9, one would expect either no change or a small decrease in overall complex enthalpy. Because DSF reports on the stability of the protein complex, and only indirectly on protein-solvent effects, the thermal stability profile reflects the van’t Hoff enthalpy (52), or enthalpy of the complex (45,53). This is in contrast to calorimetry, which reports on protein and solvent as a system. In our DSF data, AcrIF9 bound Csy had a similar temperature of maximum intensity and melting profile as unbound Csy (Figure 4), indicating that the enthalpic contribution to stability is similar. Taken together, the HDX and DSF data support a model in which binding of AcrIF9 to the Csy complex is entropically driven. To further test this model, we also performed isothermal titration calorimetry (ITC) experiments to measure the energetics associated with AcrIF9 binding to the Csy complex (Supplemental Figure S8A). Table 1 summarizes these observations which, when taken together, support the conclusion that AcrIF9 binding is an entropy driven event.

**Table 1.** Data summary

Condition	Change in HDX	Change in Tm	Binding Δ*H* (ITC)	Driving force for binding
AcrIF9	Increase	–	>0	Entropy (–*T*Δ*S*)
AcrIF2	Decrease	Increase	<0	Enthalpy (Δ*H*)
ssDNA	Decrease	Increase	<0^a^	Enthalpy (Δ*H*)

The overall trends in HDX, DSF and ITC data are summarized here for the AcrIF9, AcrIF2 and ssDNA bound Csy complexes. For the AcrIF9 bound complex, we observed an increase in deuterium exchange, no observable change in Tm and an unfavourable binding enthalpy. By combining these three observations, it can be concluded that AcrIF9 binding is an entropically stabilized event. Conversely, both AcrIF2 and ssDNA binding are enthalpically stabilized events as confirmed by decreased deuterium exchange, an increase in Tm, and a favourable enthalpy of binding.

^a^This conclusion is derived from DNA binding experiments performed in (8, 29).

### AcrIF2 binding is enthalpically stabilized

AcrIF2 and AcrIF9 binding to the Csy complex had opposite HDX trends. There was protection from deuterium exchange upon AcrIF2 binding in the Cas7 backbone as well as the tail of the complex (Cas5 and Cas8f, Figures 2 and 3). The impact of AcrIF2 binding, including changes to distal areas of the Csy complex, was unexpected. This decrease in deuterium exchange could be the result of a global stabilization of the hydrogen-bonding network in the AcrIF2 bound form of Csy. This implies a more favorable enthalpy, meaning stronger non-covalent interactions. A global increase in the stability of the hydrogen bonding network would limit the conformational freedom of the complex and cause a concomitant decrease in entropy.

If AcrIF2 binding does lead to a more favorable overall enthalpy, one would predict an increase in the thermal stability of the complex. The melting curve of AcrIF2 bound Csy is shifted to a higher temperature than that of Csy by about 3°C (Figure 4). This is consistent with stronger non-covalent interactions within the complex and a more negative enthalpy of binding. A greater enthalpic contribution to AcrIF2 binding is supported by the HDX data where there was less exchange (longer-lived hydrogen bonding networks) in the AcrIF2 bound complex. Taken together, this data favors a model in which binding of AcrIF2 is an enthalpically stabilized reaction (Table 1). This model is also consistent with results from ITC (Supplemental Figure S8B).

### Binding of Acrs alters the conformational landscape implying a role for allostery

In our HDX data, we observed changes in deuterium exchange throughout the Csy complex when either Acr was bound. These global changes demonstrate that the hydrogen bonding network is altered throughout the complex, which implies that the conformational landscape was modified upon Acr binding. The conformational landscape of a protein is a representation of the ensemble of conformations present at any given time, with the lowest energy conformations being the most populated (37,54,55). The landscape of the ligand free protein contains the ligand bound conformations; however, without ligand present they may be at a higher energy and thus infrequently populated (37). By monitoring changes in the hydrogen bond network of the Csy complex bound to AcrIF9 or AcrIF2, we were essentially tracking differences in the conformational ensemble and therefore the energy landscape. In the AcrIF9 bound Csy complex, we propose increased conformational freedom implying that there are a series of bound conformations with similar energy. In the AcrIF2 bound Csy complex, the HDX and DSF results suggest a stabilized hydrogen bond network, thus the favored ensemble would collapse into a limited set of conformations. The remodeling of the energy landscape upon either Acr or ssDNA binding, emphasizes the difference in conformational freedom available to each bound complex. This conformational freedom, or lack thereof, may present itself as changes in dynamics upon ligand binding.

The global changes in protein dynamics may have further implications than simply stabilizing the Acr–Csy interactions. The observation that there are changes in HDX distal to either of the Acr binding sites, indicates there is an allosteric network within Csy (56), which is in congruence with allosteric networks observed in other Type I interference complexes (35). The binding of either Acr alters the hydrogen bonding network, resulting in global changes to Csy. While the structural changes are not large, there are distinct functional outcomes. For example, either AcrIF9 or AcrIF2 bound Csy will no longer bind dsDNA specifically. We posit that the functional outcomes may have more to do with the altering the thermodynamic profile of Csy, than trapping a novel conformation. This alteration in the absence of overt conformational change, can be indicative of an allosteric network (55). Communication within this network begins at the binding site of either Acr and spreads from the tail to the head of the complex. This network may play a role in each ligand interaction with the Csy complex. Allosteric changes upon dsDNA binding are also present in the type IE interference complex Cascade (35). This suggests that allosteric networks play a significant role in the function of type I CRISPR interference complexes.

### AcrIF9 interactions with DNA

Allosteric effects have an impact on the ability of Csy to bind nucleic acids. In the AcrIF2 bound Csy complex, DNA interactions are blocked. However, AcrIF9 bound Csy can still interact with dsDNA through non-specific interactions and ssDNA through sequence specific binding (57,58). The specificity of ssDNA binding implies that it binds directly to the crRNA template (57), while non-specific binding of dsDNA to the AcrIF9 bound Csy complex occurs mainly through interactions with the two Acrs, as well as the N-terminal hook of Cas8f (58). Because AcrIF9 does not bind dsDNA on its own, there must be a conformational change upon binding to Csy that increases affinity for dsDNA or there is an inherent low affinity that is only observable with multi-valent binding. Interestingly, the non-specific dsDNA binding is not limited to only AcrIF9–Csy complexes. In Liu *et al.*, it was observed that AcrIF14 (which also interacts with Cas7.4f and Cas7.6f) induces non-specific dsDNA binding when complexed with Csy, though the mechanism of this binding differs from that of non-specific dsDNA binding to the AcrIF9–Csy complex (59).

**Figure 5.**
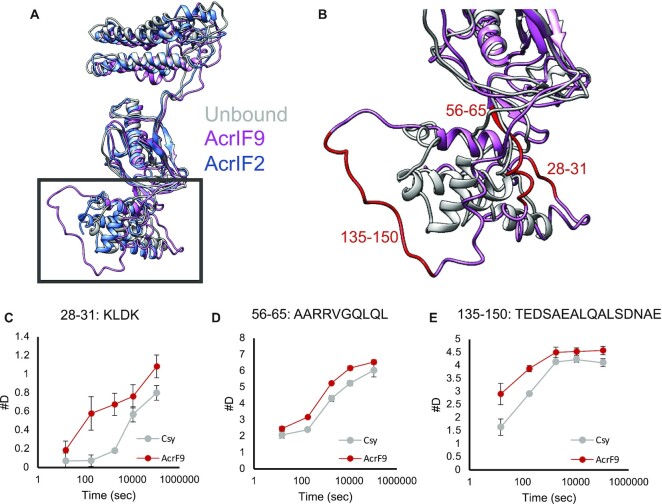
AcrIF9 changes the conformation and deuterium exchange in the Cas8f N-terminal hook region. (**A**) Alignment of Cas8f unbound (PDBID: 6B45, white), AcrIF2 bound (PDBID: 6B47, blue), and AcrIF9 bound (PDBID: 6VQV, pink). (**B**) A closer view of the N-terminal hook (amino acid residues 1–166) comparing the AcrIF9 bound structure (pink) to the unbound structure (grey). In this region three peptides had increased deuterium exchange in the AcrIF9 bound complex (red). (**C–E**) Uptake curves showing the deuterium uptake for peptides that show differences in exchange in the N-terminal hook. Each data point represents the average of three replicates with error bars representing the standard deviation of those three replicates.

In both types of DNA binding, changes in the Cas8f subunit upon AcrIF9 binding play a role. In non-specific dsDNA binding, interactions between dsDNA and Cas8f may be facilitated through an increase in solvent accessibility or protein dynamics. HDX shows that the Cas8f N-terminal hook has areas of increased exchange upon AcrIF9 binding in both fast (seconds) and slow (minutes) exchanging time points (peptides 28–31, 56–65, 135–150, Figure 5). These patterns of exchange are in concordance with the respective structural models (Figure 5B) (10,38). In the intact HDX data, there is an increase in exchange in Cas8f when AcrIF9 is bound at the early to middle time points (1–60 min, Figure 2). The increase is most pronounced at the 8-minute time point, suggesting enhanced dynamics or conformational change. This change may inhibit the ability of Cas8f to productively search for a PAM sequence or to perform strand separation and therefore prevent the AcrIF9 bound complex from binding dsDNA with sequence specificity. Taken together, the changes that occur in Cas8f upon AcrIF9 binding are likely to be involved with Csy inhibition. Because AcrIF9 does not bind in the N-terminal hook region, control must be through an allosteric network.

## CONCLUSION

The biophysical analysis of anti-CRISPR binding to the Csy complex revealed distinct perturbations to the conformational landscape that were inhibitor specific. The two Acrs tested provide a striking example of the mechanistic diversity and thermodynamic driving forces that facilitate biomolecular interactions. Upon AcrIF2 binding in the tail, we observed a stabilization of the hydrogen bonding network in the Cas8f subunit and along the entire Cas7 backbone. Comparisons with the ssDNA bound complex, shows that there is a similar stabilization of Cas8f in both conditions; however, stabilization of Cas7 is more pronounced once AcrIF2 binds than it is after binding ssDNA (Figure 2). This suggests that AcrIF2 binding in the tail, not only obscures the PAM sensing domain, but it also stabilizes the compressed conformation of the Cas7 backbone.

In the AcrIF9 bound form of the complex, we observed changes in deuterium uptake which indicate that the hydrogen bonding network is perturbed. This implies that the conformational landscape is remodeled such that the lowest energy conformation has a more dynamic Cas7 backbone (Figure 2). This could introduce an insurmountable entropic penalty to specific dsDNA binding. Anti-CRISPRs are diverse and most-likely have evolved independently of each other, which explains the mechanistically distinct action. From an evolutionary standpoint, it makes sense that anti-CRISPRs would take advantage of entropically and enthalpically driven inhibition. While there are examples of entropically driven regulation of protein complexes (55,60), it is less commonly reported. This could stem from the more challenging nature of capturing changes in entropy. We posit that the thermodynamically distinct mechanisms evident in anti-CRISPR mediated inhibition of the Csy complex are not an extraordinary example of viral evolution, but rather a theme that is common in the regulation of protein complexes across all domains of life.

## DATA AVAILABILITY

The mass spectrometry proteomics data have been deposited to the ProteomeXchange Consortium via the PRIDE partner repository with the dataset identifier PXD036446. Protein structural data can be found in the Protein Data bank (https://www.rcsb.org/) under PDB codes: 6B44, 6B47, 6B45 and 6VQV.

## Supplementary Material

gkac841_Supplemental_Files

## Notes

Present address: Elizabeth Waymire, Biology Department, Baylor University, Waco, TX 76798, USA.
