# Supplementary material for: Anti-CRISPR proteins function through thermodynamic tuning and allosteric regulation of CRISPR RNA-guided surveillance complex
Source: Nucleic Acids Res. 2022 Oct 10;50(19):11243–54. doi: 10.1093/nar/gkac841 (PMC9638894; doi:10.1093/nar/gkac841)
Supplement: gkac841_Supplemental_Files [file gkac841_supplemental_files.zip › Revised_Supplementary_Material-HDX of Csy and Acrs_2022_0908.pdf]

Supplementary Information

Anti-CRISPR proteins function through thermodynamic tuning and allosteric regulation of CRISPR RNA-guided surveillance complex

Angela Patterson[1], Aidan White[1], Elizabeth Waymire[1], Sophie Fleck[1], Sarah Golden[2], Royce Wilkinson[2], Blake Wiedenheft[2], Brian Bothner[1,*]

[1] Chemistry and Biochemistry Department, Montana State University, Bozeman, MT, 59717, USA

[2] Microbiology and Cell Biology Department, Montana State University, Bozeman, MT, 59717, USA

[*]Correspondence to: bbothner@montana.edu

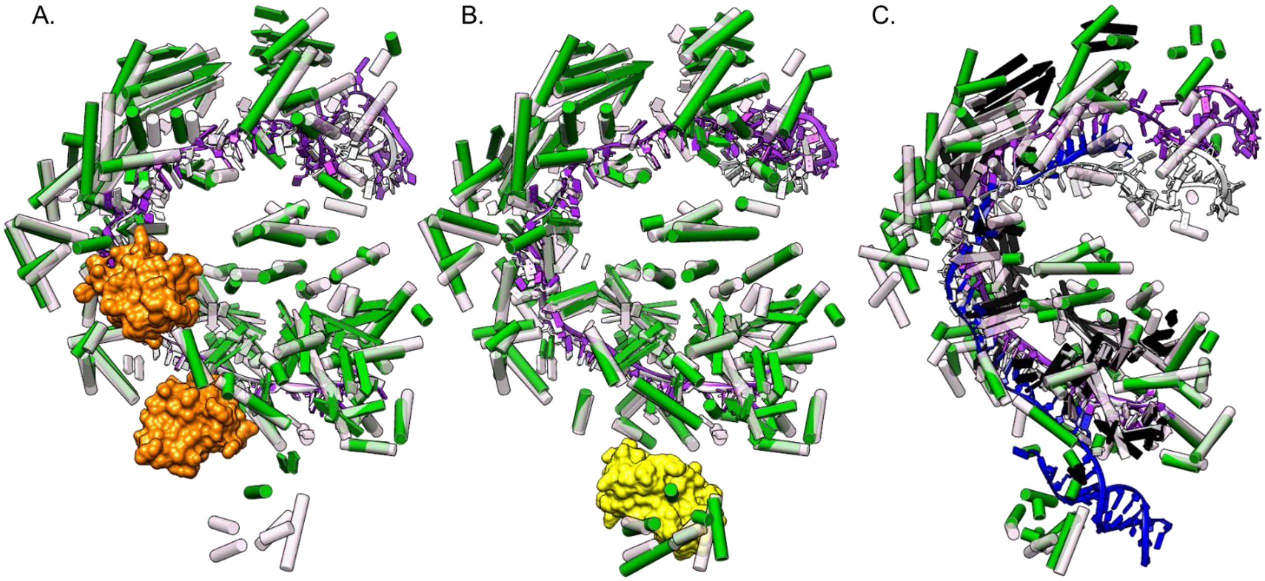

Supplemental Figure 1. Structural comparison of Csy forms. Structure of unbound Csy is white in each panel (PDB ID:6B45 obtained through Cryo-EM at a resolution of 3.5Å). A) AcrIF9 (green complex, PDB ID:6VQV obtained through Cryo-EM at a resolution of 2.57Å, Cas8 based alignment with a RMSD 2.892). B) AcrIF2 bound Csy (green complex, PBD ID:6B47 obtained through Cryo-EM at a resolution of 3.20Å, Cas8 based alignment with a RMSD 5.812). C) dsDNA bound (green complex, PDB ID:6B44 obtained through Cryo-EM at a resolution of 2.9Å, Cas8 based alignment). AcrIF9 is displayed in orange, AcrIF2 in yellow and dsDNA in bright blue. crRNA is shown in purple.

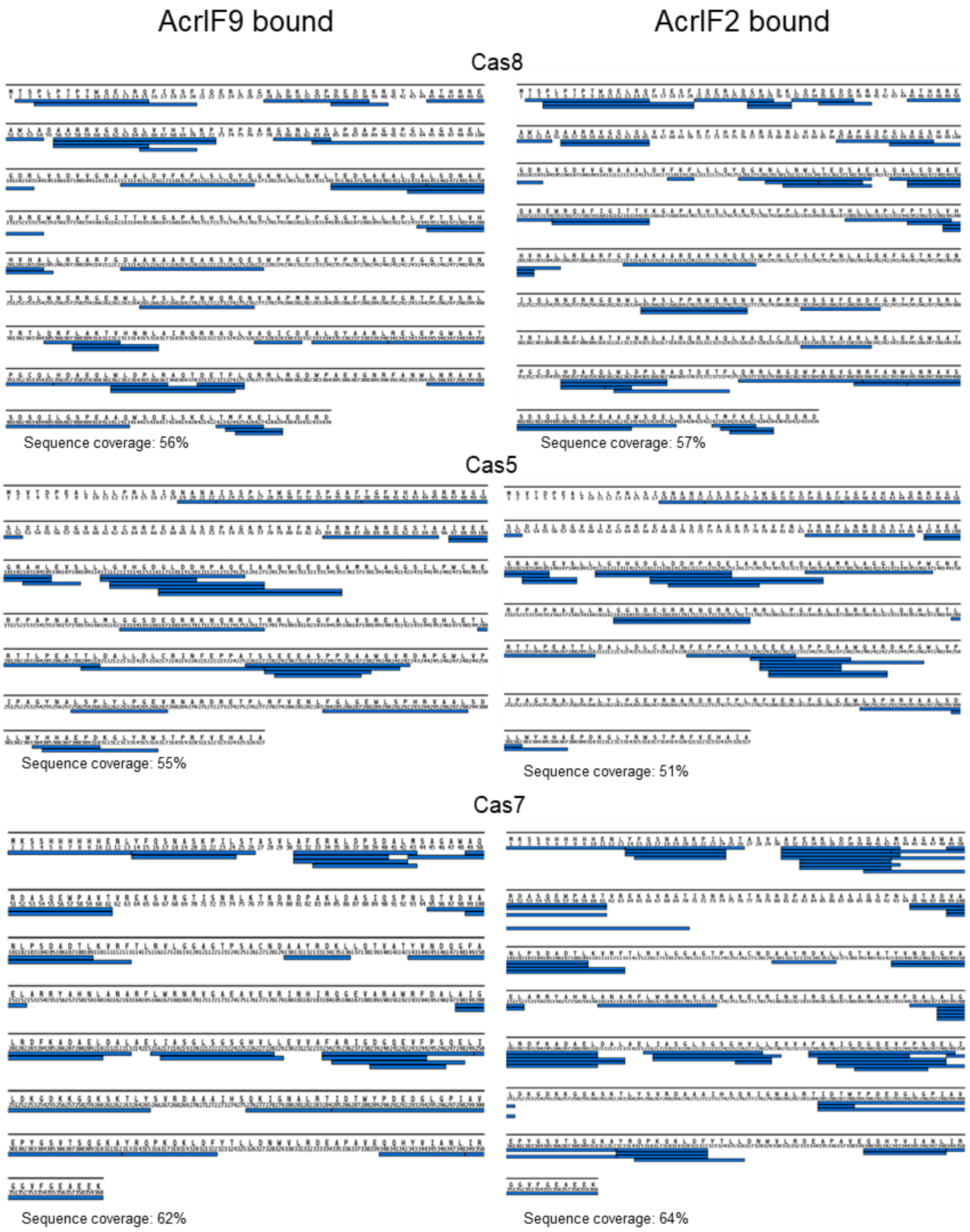
AcrIF9 bound
AcrIF2 bound
Cas8
Sequence coverage: 56%
Sequence coverage: 57%
Cas5
Sequence coverage: 55%
Sequence coverage: 51%
Cas7
Sequence coverage: 62%
Sequence coverage: 64%

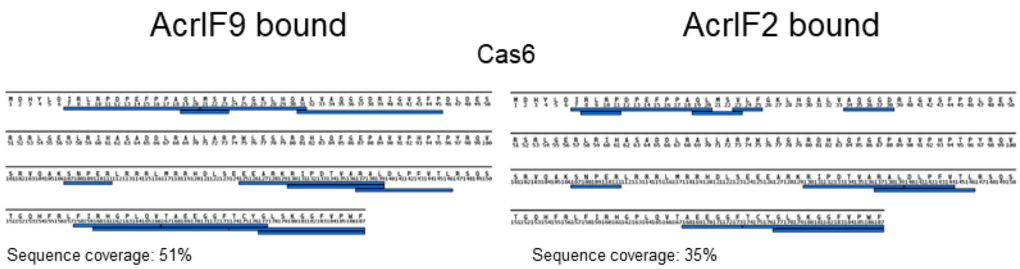

Supplemental Figure 2. Peptide maps for each subunit in either the AcrIF9 bound condition or the AcrIF2 bound condition. To ensure unambiguous assignment of peptides, a stringent selection process was applied (see methods for details). The final maps for the Csy complex in the AcrIF9 bound condition covered 741 residues (57%) and 710 residues for the AcrIF2 bound condition (54%). Only peptides showing unimodal deuterium uptake behavior are mapped onto the structure in Figure 2 and shown in supplemental figure 3 butterfly plots.

Cas5

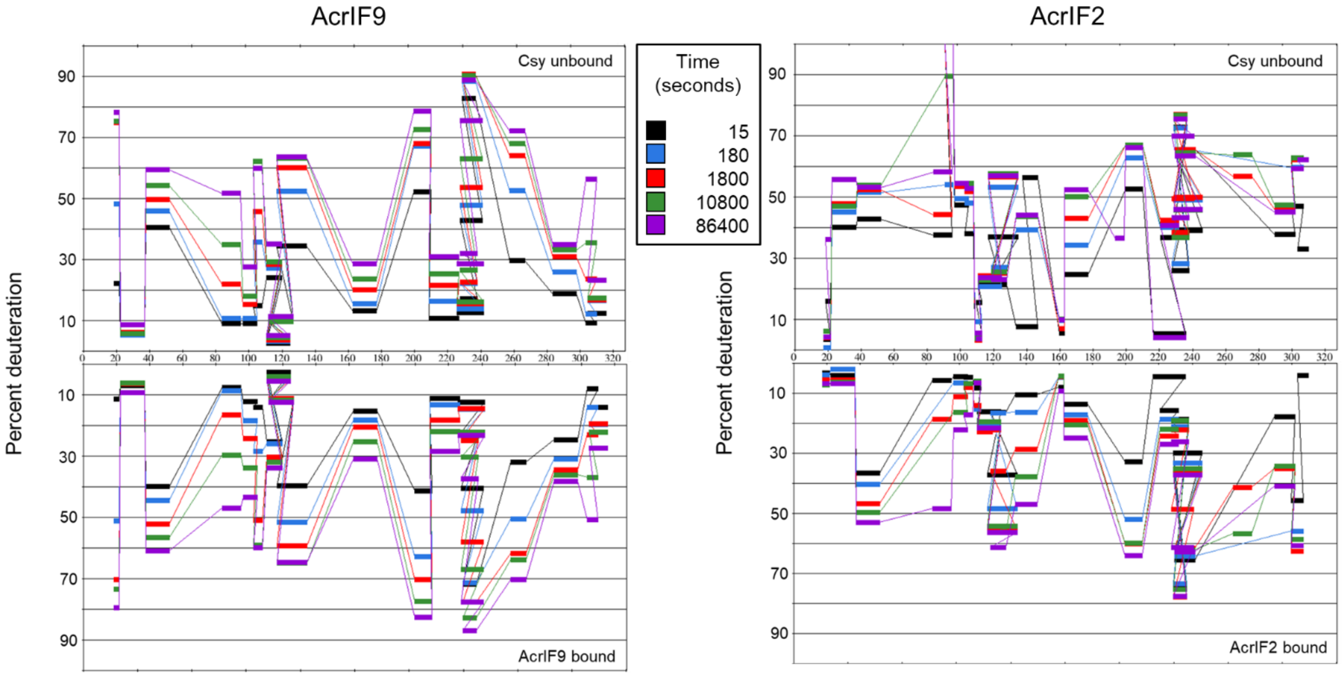
AcrIF9
AcrIF2
Csy unbound
Csy unbound
Time (seconds)
15
180
1800
10800
86400
Percent deuteration
Percent deuteration
AcrIF9 bound
AcrIF2 bound

Cas6

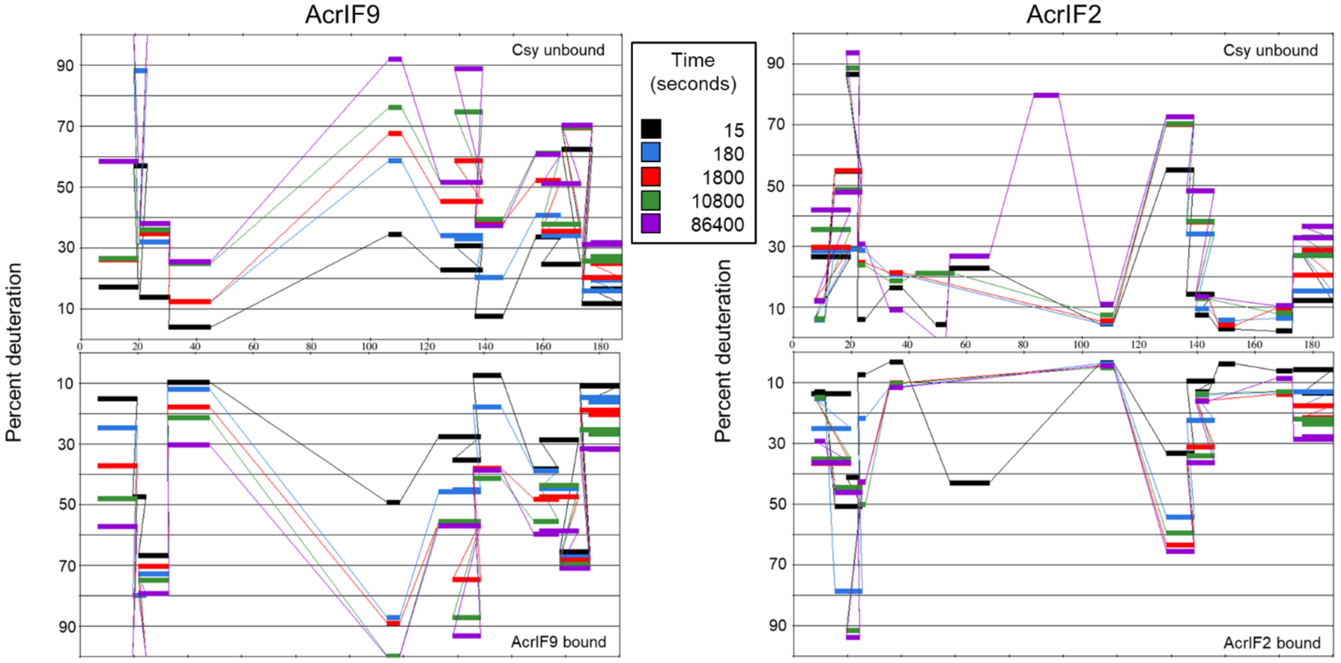
AcrIF9
AcrIF2
Csy unbound
Csy unbound
Time (seconds)
15
180
1800
10800
86400
Percent deuteration
Percent deuteration
AcrIF9 bound
AcrIF2 bound

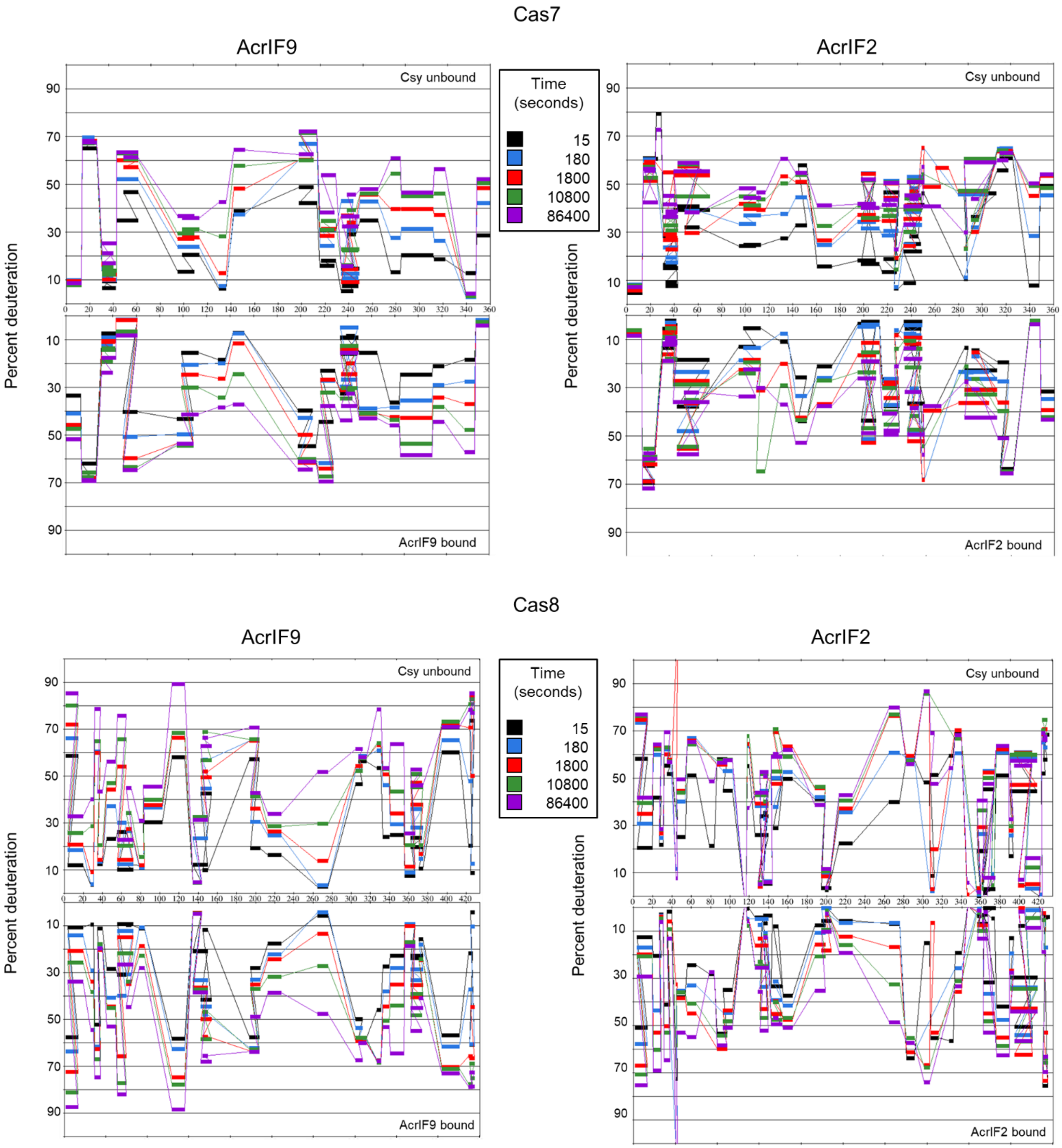

Supplemental Figure 3. Butterfly plots showing percent deuteration vs amino acid residue number of the peptide level HDX for each indicated subunit. The comparison of Csy unbound (top) and AcrIF9 bound (bottom) are displayed on the left. The comparison of Csy unbound (top) and AcrIF2 bound (bottom) are displayed on the right. Each time point is represented by a different color line as indicated in the middle color key. Percent deuteration was calculated by taking the observed deuterium uptake and dividing by the maximum theoretical uptake (number of residues present in the peptide not including the two n-terminal residues or proline).

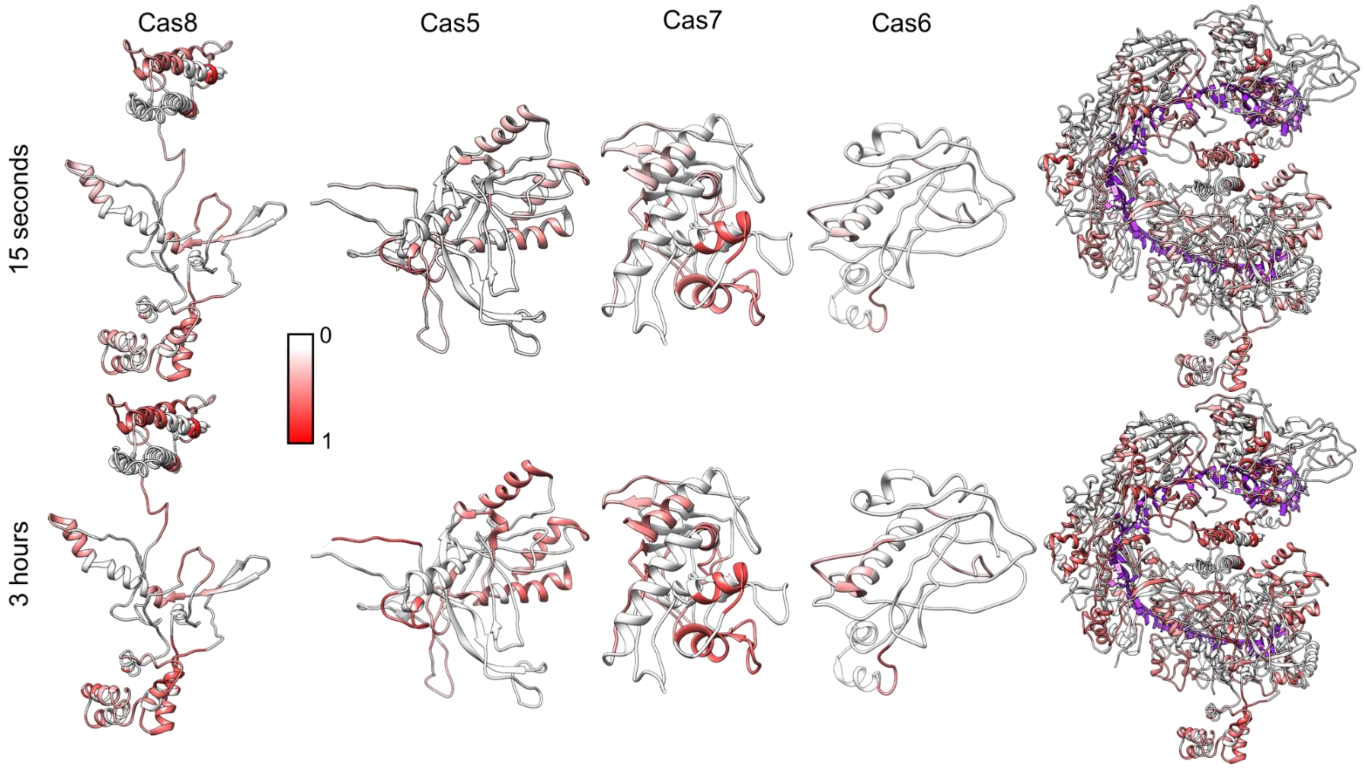

Supplemental Figure 4. Structural mapping of the unbound Csy peptide deuterium uptake at 15 seconds and 3 hours. The peptide level uptake is displayed on each subunit of the Csy complex (Cas7.1 was used to represent the Cas7 subunits) as well as on the complex as a whole. The orientation of the subunits were used constant throughout the figure. The amount of deuterium taken up per residue within a peptide is displayed as a heat map where red represents one deuterium per residue and white indicates no deuterium uptake or no coverage. To map the uptake onto the structure only peptides that could be resolved (contained a common N or C-terminus) were used. If a peptide was represented by multiple charge states, then the uptake was averaged.

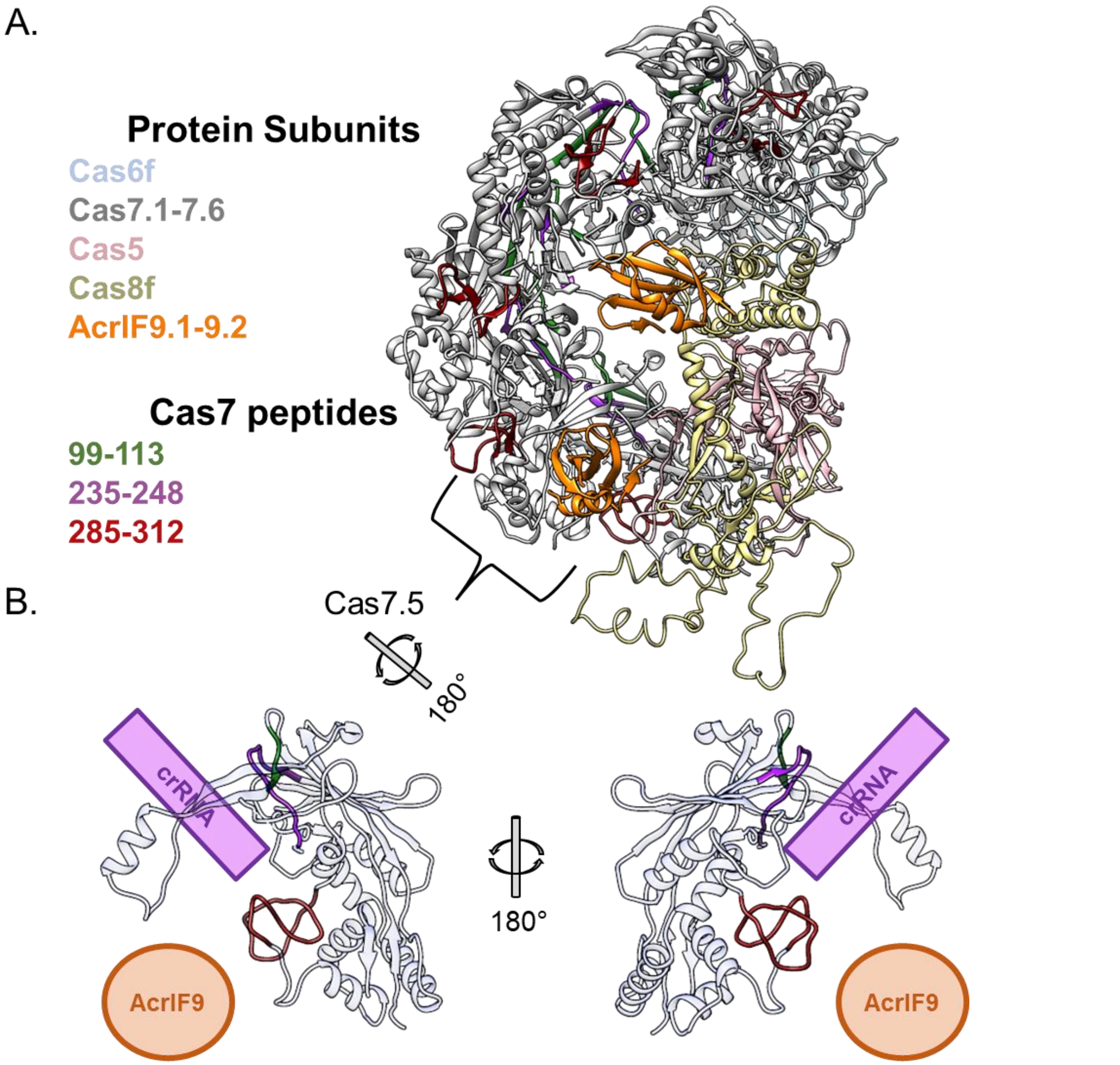

Supplemental Figure 5. Location of Cas7 bimodal peptides when AcrIF9 is bound to the Csy complex. There are six copies of Cas7f within the Csy complex which leads to multiple different environments that can affect peptide dynamics within the Cas7 population. A) The three peptides that exhibited clear bimodal behavior were mapped to the complex structure: 99-113 (dark green), 235-248 (purple), and 285-312 (dark red). B) A rotated close-up view of Csy subunit Cas7.2 in relation to the crRNA and AcrIF9.2. Peptide coloring is the same as panel A. While none of these peptides are located in the AcrIF9:Csy interface (closest side chain distances between red peptide and AcrIF9 are >15Å and peptide 285-312 is shielded from interactions with AcrIF9 by the loop containing residues 70-94 of Cas7.6), they are within 5 Å of Cas5 in the Cas7.6 subunit and in the Cas7.1 subunit, residues 285-312 come within 5 Å of the Cas7.1:Cas6f interface. The bimodal behavior of these peptides can be explained by different environments at the head or the tail of the Csy complex. Structural model is based on PDBID: 6VQV.

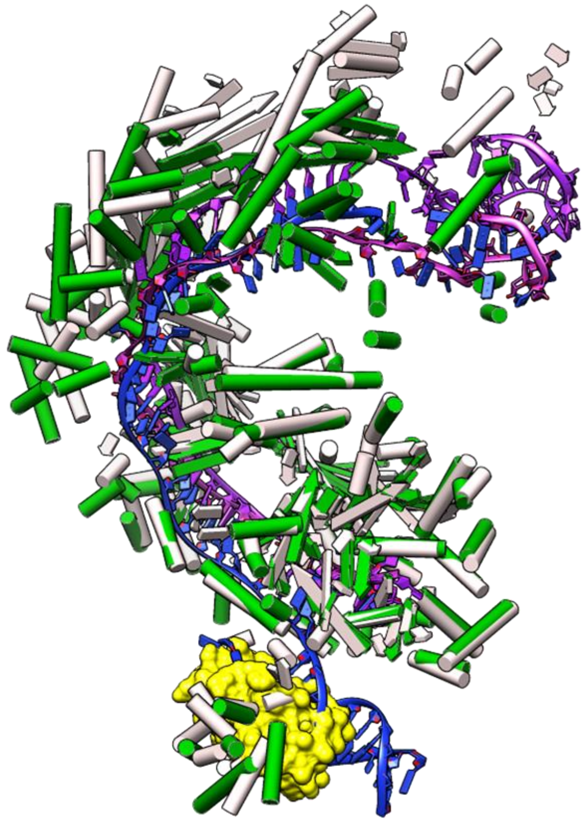

Supplemental figure 6. Comparison between dsDNA bound Csy and AcrIF2 bound Csy. The dsDNA bound structure (PDBID 6B44, white with dsDNA in bright blue) was aligned to the AcrIF2 bound structure (PDBID 6B47, green, AcrIF2 in yellow).

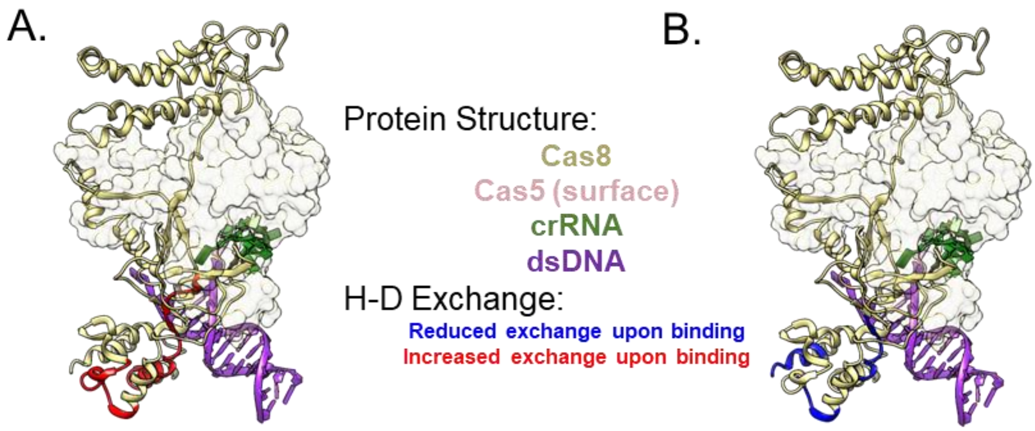

Supplemental figure 7. Location of mentioned peptides that show differences in deuterium exchange upon ssDNA binding within the Cas8 subunit. A. The peptides that showed an increase in deuterium exchange (red, amino acid residues 66-72, 135-150). B. The peptides that show a decrease in deuterium exchange both in the ssDNA bound condition and in the AcrIF2 bound condition (blue, 4-20, 56-65, 145-150). PDBID: 6B44. The peptide spanning residues 135-150 showed an increase in deuterium exchange while the overlapping peptide containing residues 145-150 showed a decrease in deuterium exchange. Because these two peptides share a C-terminal residue, we can resolve the exchange and conclude that residues 135-145 show an increase in exchange that is larger in magnitude than the protection from exchange that is observed for residues 145-150.

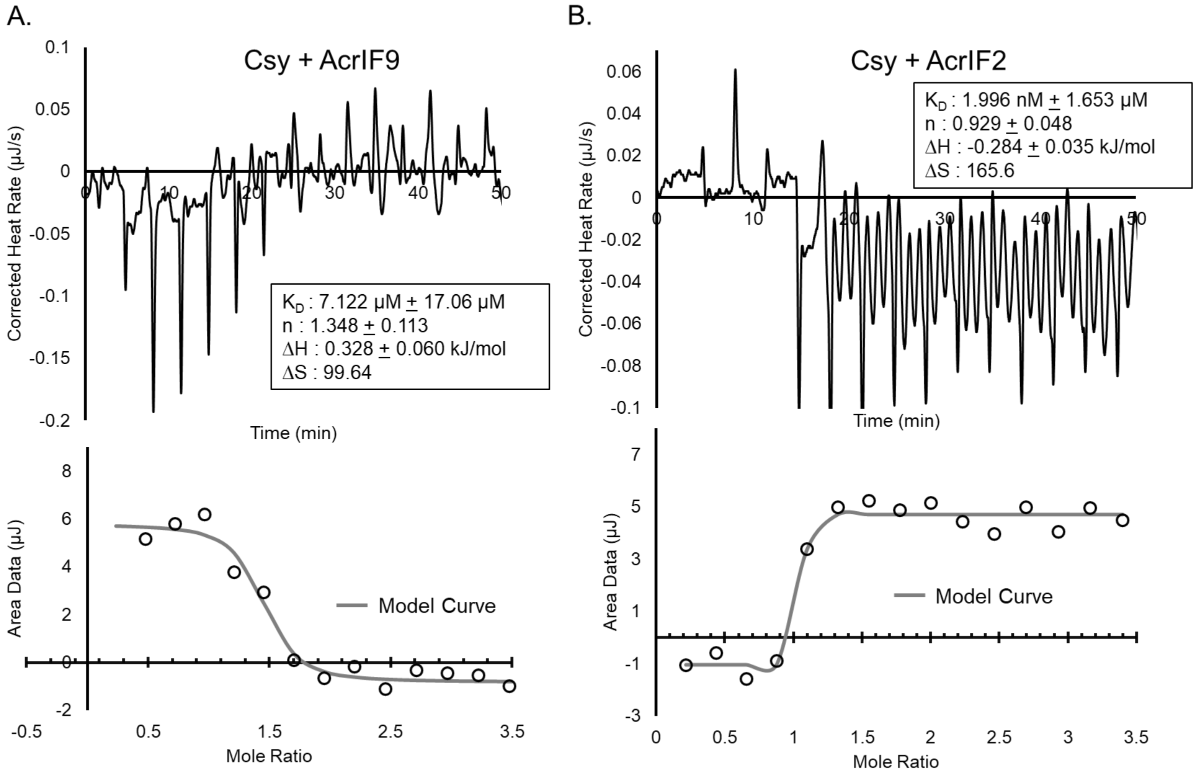

Supplemental figure 8. Isothermal titration calorimetry (ITC) curves for the investigation of thermodynamic parameters of Acr binding. Top curves show the ITC plot of corrected heat rate vs time for AcrIF9 (A, top) and AcrIF2 (B, top) binding to the Csy complex. Values above zero indicate an exothermic reaction while those below zero indicate an endothermic reaction. The calculated area of each peak is displayed in the lower panels (AcrIF9, A, bottom; AcrIF2, B) plotted against the mole ratio of Acr to Csy complex. The grey line indicates the fit of the model used to determine the thermodynamic parameters displayed in the box.

For the ITC experiments either AcrIF9 or AcrIF2 were adjusted to 20µM using post-column SEC buffer (20 mM tris, pH 7.5, 100 mM NaCl, 1mM TCEP, 5% glycerol). The Csy complex was adjusted to 0.5µM using the post-column SEC buffer. ITC was performed using a Nano ITC (TA Instruments) with a tank volume of 170µL set at 25°C. The Acr was set as the titrant and 1µL was delivered every 200 seconds. Stir speed was set to 270 rpm. Data were analyzed using NanoAnalyze Data Analysis (TA Instruments, v3.12.5). Modeling for determination of thermodynamic parameters was performed using the Blank (constant) and Independent model features included in the software package.
